# Analytical approximations for spatial stochastic gene expression in single cells and tissues

**DOI:** 10.1098/rsif.2015.1051

**Published:** 2016-05

**Authors:** Stephen Smith, Claudia Cianci, Ramon Grima

**Affiliations:** School of Biological Sciences, University of Edinburgh, Mayfield Road, Edinburgh EH9 3JR, UK

**Keywords:** chemical master equation, spatial modelling, noise, gene expression, stochastic models

## Abstract

Gene expression occurs in an environment in which both stochastic and diffusive effects are significant. Spatial stochastic simulations are computationally expensive compared with their deterministic counterparts, and hence little is currently known of the significance of intrinsic noise in a spatial setting. Starting from the reaction–diffusion master equation (RDME) describing stochastic reaction–diffusion processes, we here derive expressions for the approximate steady-state mean concentrations which are explicit functions of the dimensionality of space, rate constants and diffusion coefficients. The expressions have a simple closed form when the system consists of one effective species. These formulae show that, even for spatially homogeneous systems, mean concentrations can depend on diffusion coefficients: this contradicts the predictions of deterministic reaction–diffusion processes, thus highlighting the importance of intrinsic noise. We confirm our theory by comparison with stochastic simulations, using the RDME and Brownian dynamics, of two models of stochastic and spatial gene expression in single cells and tissues.

## 1. Introduction

The biochemical processes of transcription and translation involve species which exist in very low concentrations [1–5]. In these cases, intrinsic noise does not average out, and hence stochastic effects are important [6–9]. Although these effects are highly significant to cell physiology, they cannot be described by the well-known rate equations (REs) which are generally accurate *in vitro*. Mathematical modelling of these systems has correspondingly changed its focus towards more detailed non-spatial stochastic approaches based on the chemical master equation (CME) [10–13]. However, these approaches implicitly assume fast diffusion, whereas experiments show that intracellular diffusion of molecules can be slow compared with *in vitro* [14] and thus limit the rates of many biochemical reactions. The importance of such effects has been recently demonstrated in a theoretical study of the response of an MAPK pathway [15]. Mathematical modelling of stochastic chemical systems incorporating spatial effects remains in its infancy, and little is known in comparison with stochastic systems which are well mixed. The slow development of this area can be explained by the stark difference in computational complexity between stochastic simulation algorithms (SSA) for the CME, such as the Gillespie algorithm [16–18], which models only the total number of molecules in a compartment, and the corresponding spatial algorithms such as Brownian dynamics (BD) [19], which additionally explicitly model particle positions over time. Furthermore, the lack of an exact equivalent of the CME for spatial stochastic systems has made analytical approaches to diffusion generally intractable.

Here, we attempt to resolve this problem by analytically studying the reaction–diffusion master equation (RDME), an approximate description of stochastic reaction–diffusion processes [20–22]. Specifically, space is divided into a lattice of small subcompartments or ‘voxels’. Chemical reactions occur in each voxel, and diffusion occurs between neighbouring voxels. The master equation describing these processes is called the RDME. The RDME has been shown to be a good approximation to the continuum formulation of BD for specific ranges of lattice spacing and diffusion coefficients [21], though it has also been shown that incorrect choice of lattice spacing can lead to inaccurate results [23]. Because it provides coarse-grained information about particle positions, the RDME is a trade-off between the simplicity of the CME and the fine-grained accuracy of BD. The RDME is also an appropriate description of the dynamics of a tissue of intercommunicating cells when each cell is under well-mixed conditions.

Our approach to analytically studying the RDME is based on a recently developed technique known as effective mesoscopic rate equations (EMREs) [24]. This technique has been used to obtain approximate formulae for mean molecule numbers in CME models. In particular, these formulae have been shown to accurately capture the differences between the mean protein numbers calculated using the CME and the RE [13,25]. We here adapt and apply the EMRE approach to the RDME of a general biochemical system and thereby derive spatial effective mesoscopic rate equations (sEMREs). The sEMRE is a general method that approximates the mean concentrations of chemical species in a reaction–diffusion system. In the special case of systems with a single chemical species, we can obtain closed-form expressions for the sEMRE which are useful for investigating the dependence of mean concentrations on diffusion rates. We subsequently apply our novel theory to obtain closed-form expressions for the approximate steady-state protein mean concentrations in two models of spatial gene expression in single cells and in tissues, as well as an example that further models the effect of molecular crowding. These expressions show a dependence on the diffusion coefficients which is not captured by the classical deterministic reaction–diffusion theory. We test our formulae against RDME and BD simulations and show good agreement over a range of diffusion coefficients.

## 2. Approximate equations for mean concentrations of non-spatial chemical systems

### 2.1. Rate equations

**Figure 1.**
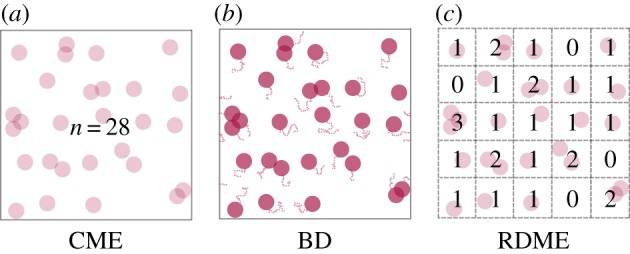
An illustration of how the CME and RDME approximate the underlying BD process. (*b*) BD consists of a set of particles with fixed radii (red circles) performing a random walk (dotted tails) in continuous space. Particles react with a given probability when their radii are overlapping. (*a*) The CME loses all spatial resolution and models only the total number of molecules *n*, in this case *n* = 28. The faded particles illustrate only that the CME models an underlying spatial process (BD), even though the CME itself does not consider particles in space. (*c*) The RDME achieves coarse-grained spatial resolution by introducing a spatial grid, in this case a 5 × 5 grid. Inside each grid square (voxel), only the total number of particles is modelled (analogously to the CME), whereas the detailed location of particles inside voxels is ignored. Bimolecular reactions can happen only if a voxel contains at least two reacting particles. Diffusion occurs by particles hopping between neighbouring voxels.

In this section, we briefly review the deterministic RE approach which consists of a set of coupled ODEs whose solution approximates the time evolution of the mean concentrations of the CME, and which is valid in the limit of large molecule numbers. The relationship between the CME and BD is illustrated in figure 1. We describe the approach on a generic system of reactions, as follows. Consider a system of *M* chemical species involved in *R* reactions, where the *j*th reaction has the form
2.1
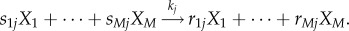
Here, *X_i_* denotes the chemical species, *s_ij_* and *r_ij_* are the integer stoichiometric coefficients and *k_j_* is the reaction rate constant for reaction *j*. The CME for this system is defined by the following equation:
2.2
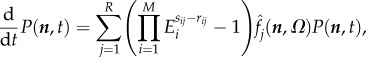
where *Ω* is the volume in which the reactions occur, ***n*** = (*n*_1_, … ,*n_M_*) is a vector of the number of molecules of *X*_1_, … ,*X_M_,* respectively; *P*(***n***, *t*) is the probability of finding the system with ***n*** copies of each species at time *t*, 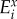 is an operator which replaces *n_i_* with *n_i_* + *x*, and 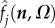 is the microscopic propensity function of reaction *j*, which takes the form 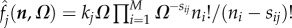 under mass-action kinetics. The mean number of molecules of *X_i_* at time *t* is given by the usual expected value
2.3
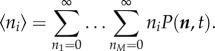

While equation (2.3) can theoretically be combined with equation (2.2) to obtain ODEs for 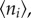 the resulting equations cannot, in general, be solved exactly, and moment-closure techniques must be used [26]. Alternatively, it can be shown that a large volume expansion of the CME leads to the result
2.4
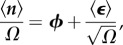
where 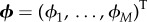 is a vector of deterministic concentrations of species 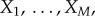 respectively, 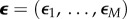 is a continuous random vector [27] of fluctuations about the deterministic concentration and 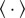 denotes expected value. The vector of deterministic concentrations ***ϕ*** is the solution of the well-known rate equations
2.5
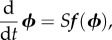
where 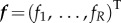 is the macroscopic rate vector, and *f_j_* is the macroscopic reaction rate of reaction *j*, which takes the form 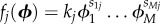 under mass-action kinetics. Other forms of reaction rates exist such as Hill-type and Michaelis–Menten (MM), and we discuss such an example in §5. Note that *S* is the stoichiometric matrix with entries 
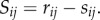


It has been shown that 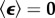 for systems with at most first-order reactions (
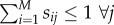
) [28] and for a subset of reversible systems (including those with bimolecular reactions) in detailed balance [29]. It follows that the RE solution ***ϕ*** is exactly equal to the mean concentrations 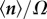 for these systems. For other systems, 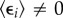 and so estimating the expected value is essential to computing accurate mean concentrations.

### 2.2. Effective mesoscopic rate equations

The first-order approximation to 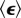 is given by a set of ODEs called EMREs (originally derived in [24]). The time-evolution equation for 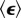 is
2.6
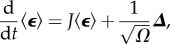
where 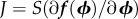 is the Jacobian of the deterministic REs, and 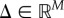 is a vector whose *i*th element is defined as
2.7
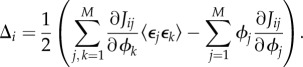


The covariance 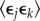 can be computed as the (*j*, *k*)th element of the matrix 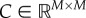 which solves the Lyapunov equation
2.8
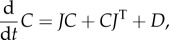
where 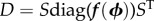 is the diffusion matrix. Note that the covariance of fluctuations in molecule numbers of two species *X_i_* and *X_j_* is 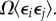 Hence, the estimate of the mean concentration using the EMRE takes into account, via the vector ***Δ***, the coupling between the mean and the covariance of fluctuations. Note that the vector ***Δ*** is only non-zero if the Hessian of the REs is non-zero and hence a necessary (but not sufficient) condition for 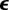 to be non-zero is that the system is composed of at least one reaction with a nonlinear reaction law, such as a bimolecular reaction. Note that equation (2.7) is only valid for a system of elementary reactions (input, unimolecular and bimolecular); a generalization to the case where some of the reactions are non-elementary can be found in appendix C.

The EMRE itself is a time-evolution equation for the approximate mean concentrations ***ψ***, which is defined as 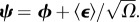 The defining equation for ***ψ*** is obtained by substituting equation (2.4) into equation (2.6)
2.9
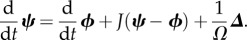


In steady state, all time derivatives are zero, so we recover the simpler equations for the EMRE
2.10
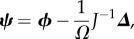
and the steady-state Lyapunov equation
2.11
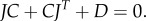


For a system consisting of only one chemical species *X*, the EMRE simplifies dramatically. The reaction system can be written as
2.12
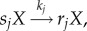
for *j* = 1, … , *R*, for stoichiometric coefficients *s_j_* and *r_j_*. The stoichiometric matrix *S* will, in this case, be a stoichiometric vector with entries *S_j_* = *r_j_* − *s_j_*, and the mass-action rate vector 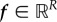 will have elements defined as 
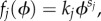
 where *ϕ* is now the steady-state deterministic concentration of *X*.

Because this is a single-species system, the Jacobian and diffusion matrices will simply be real numbers, *J* = *α* and *D* = *β* respectively. These are defined as
2.13
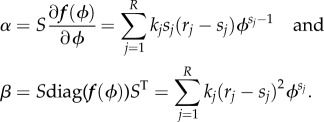
Note that stable systems must have *α* < 0, because *α* is the eigenvalue of the Jacobian, whereas *β* ≥ 0 is guaranteed by its definition. The matrix of covariances, *C*, is now simply a real number corresponding to 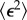 and its value can be found by solving equation (2.11) to find 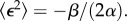 Similarly, the vector ***Δ*** is now a scalar defined as 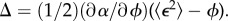 The single-species EMRE in steady-state conditions is therefore given by inserting these values into equation (2.10)
2.14
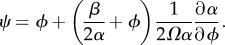
Note that the EMRE solution is given by a sum of the RE solution *ϕ* and a correction which is inversely proportional to the system size *Ω*. This result can be shown to be accurate to order *Ω*^−1^; higher-order corrections can also be calculated using the system-size expansion and have been done [30], but we shall not consider them here.

## 3. Approximate equations for mean concentrations of spatial chemical systems

### 3.1. Spatial rate equations

Just as the REs provide a deterministic approximation of the CME, one can write spatial REs which are a deterministic approximation of the RDME. To provide spatial resolution, the RDME divides space into compartments called ‘voxels’ and uses a CME-like model for each voxel. The relationship between the CME, the RDME and BD is illustrated in figure 1. In this paper, we will consider a two-dimensional *N* × *N* grid in a space of size *Ω*, where each voxel has an area *Ω/N*^2^. One- and three-dimensional descriptions are also possible, and formulae for these are given in appendix A. For each of our *M* species, *X_i_*, we now refer to *N*^2^ distinct species 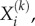 *k* = 1, … , *N*^2^, where each corresponds to *X_i_* in a different voxel. In each voxel *k,* the system undergoes *R* distinct reactions
3.1
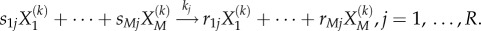
We furthermore have a set of diffusion events, which are modelled as particles hopping between neighbouring voxels. For each voxel *k,* we can define a set *Ne*(*k*) as the set of voxels neighbouring voxel *k*. The diffusion events are therefore given by the following ‘reactions’:
3.2
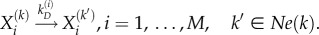
Let 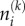 be the number of copies of 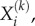 and let 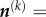 
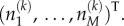
 Then, analogous to the CME in equation (2.2), we can write the RDME
3.3
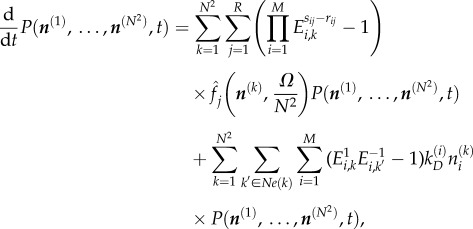
where 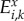 is the operator which replaces 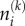 with 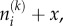 
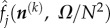
 is the microscopic rate of reaction *j*, and 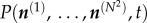 is the probability that the system is in the given state at time *t*. The first line of equation (3.3) describes the reaction system (3.1), whereas the second line describes the diffusion system (3.2). Just as in equation (2.3), we can again write the mean number of 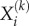 molecules as
3.4
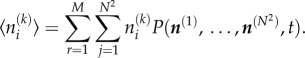
As in §2.1, this equation cannot be solved so instead we revert to the van Kampen ansatz
3.5
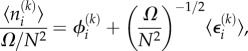
where 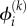 is the deterministic concentration of 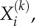 and 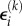 is the corresponding continuous random variable denoting fluctuations about the deterministic concentrations. Because there are *N*^2^ voxels in our system, each with four neighbours, the system in total consists of *N*^2^*R* reactions and 4 *MN*^2^ diffusion events. The vector of concentrations is 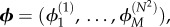 the macroscopic reaction rate vector is 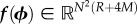 and the stoichiometric matrix has dimensions *MN*^2^ × *N*^2^(*R* + 4 *M*). The spatial REs are then defined by
3.6
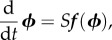
which is the spatial equivalent of equation (2.5). Note that the spatial REs are equivalent to a finite-element method for solving the well-known partial differential equations (PDEs) describing deterministic reaction–diffusion processes in continuum space. In the continuum limit of 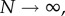 these spatial REs, therefore, become equivalent to the reaction–diffusion PDEs themselves. Note that the spatial REs are obtained from the RDME in the limit of large molecule numbers in each voxel. One way to obtain this limit is to consider the voxel size *Ω*/*N*^2^ tending to infinity while keeping concentrations constant, as can be seen from equation (3.5) (though other limits are plausible). Note, however (as we shall discuss in §4), that the choice of *N* is fundamental to the accuracy of the RDME: it should take an intermediate value that is large enough to model diffusion well, and small enough to model reactions well [23]. It follows that the spatial RE (and consequently the reaction–diffusion PDEs) have the same limitation.

Note that, in non-equilibrium conditions, the solution of the spatial REs for a single-species system is affected by diffusion. However, in steady-state conditions, provided the rate constants and diffusion coefficients are the same in each voxel, the RE solution is constant across space and precisely the same as the solution of the RE described earlier, thus implying no dependence on the diffusion coefficient. For the reaction–diffusion PDEs of a multi-species system, the effect of diffusion is given by a Laplacian operator applied to the concentrations. Because the Laplacian of a spatially homogeneous concentration is zero, it follows that the solution of the PDEs has no dependence on diffusion coefficients.

As we shall now see, just as the EMRE provides a more accurate estimate of the CME mean concentrations than the REs, so does a spatial version of the EMRE provide more accurate estimate of the means of the RDME than the spatial REs.

### 3.2. Spatial effective mesoscopic rate equation for single-species systems

This section presents the main result of this paper, namely the derivation of an approximate equation for the mean concentrations of a single-species system starting from the stochastic spatial description of the RDME. We consider the same set-up as considered for the spatial RE but for a single-species system, i.e. with *M* = 1, namely we have an effective system of *N*^2^ species and *N*^2^(*R* + 4) reactions which describe reaction and diffusion of a single species in two dimensions. We consider a single-species system, because analytical expressions can be obtained. A general derivation for multi-species systems can be found in appendix F, but such systems are analytically intractable and numerical methods must be used. We shall call the EMRE approximation applied to this system, the spatial EMRE (sEMRE). We shall also enforce the condition of spatial symmetry, introduced earlier.

By analogy with the EMRE approach, we need to first determine the *S*, *J* and *D* matrices for the spatial REs before we can obtain the sEMRE. Next, we consider in detail the construction of these matrices.

First, we consider what we can say about the Jacobian of the spatial REs of this system. Consider the diagonal element *J_ii_*, which by definition is
3.7
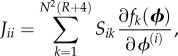
where 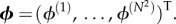 Note the lack of subscript, because we consider only a single species. For the vast majority of values of *k*, *S_ik_* = 0; the only non-zero values are those corresponding to reactions inside voxel *i*, or diffusion into and out of voxel *i*. The contribution to *J_ii_* of the internal reactions has already been calculated: it is simply *α* as defined in equation (2.13) (the symmetry of the system implies that in steady-state conditions 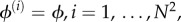 where *ϕ* is the steady-state RE solution). For diffusion *into* voxel *i*, *S_ik_* = 1 and 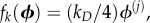 where *j* is the index of a voxel neighbouring *i* (note the factor of 1/4 is to ensure that the total rate of diffusion out of a voxel is *k_D_*). It follows that 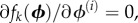 so there is no contribution to *J_ii_*. For diffusion *out of* voxel *i*, *S_ik_* = −1 and 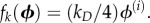 It follows that 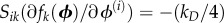 is the contribution to *J_ii_*. Because there are four distinct diffusion fluxes out of *i* (one into each neighbouring voxel), this contribution is multiplied by 4, so that 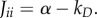 Now, consider the element *J_ij_* where *i* and *j* are neighbouring voxels
3.8
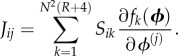
The only non-zero contributions to *J_ij_* will correspond to reactions that change the number of molecules of *X_i_* (otherwise *S_ik_* = 0) and which involve *X_j_* (otherwise, 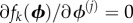), and the only reactions with this property are those describing diffusion between voxels *i* and *j*. For diffusion from *i* to *j*, 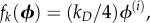 so 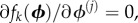 and there is no contribution to *J_ij_*. For diffusion from *j* to *i*, *S_ik_* = 1 and 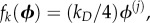 so the contribution to *J_ij_* is 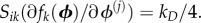 These are the only reactions contributing to *J_ij_*, so, for *j* neighbouring *i*, 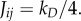


Finally, if voxels *i* and *j* are not neighbours, there are no reactions which involve both *X_i_* and *X_j_*, so the Jacobian elements are zero for these entries. In summary
3.9
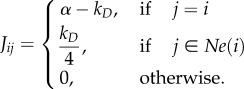
A similar argument can be used to compute the entries of the diffusion matrix *D*, which is given by
3.10
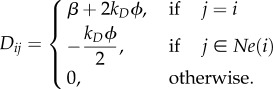
If the voxels are numbered from left to right and top to bottom, then the matrices *J* and *D* are block-circulant matrices. More details on the structure of *J* and *D* are given in appendix A. By analogy with the EMRE equation (2.10), from *J* and *D* determined above, it is possible to derive the sEMRE
3.11
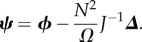
The factor *N*^2^/*Ω* appears, because each species now exists in a voxel of area *Ω*/*N*^2^. The *i*th entry of ***Δ*** is defined as in equation (2.7) (with *M* replaced by *N*^2^, because the latter is the number of effective species), but because the only entries of *J* which have any *ϕ*-dependence are the diagonal entries, this can be simplified to 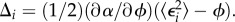 By the condition of spatial symmetry, all the 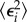 must be the same, say, 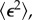 which implies that the vector **Δ** can be simplified to 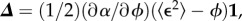 where 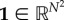 is a column vector of 1 s.

The sEMRE is then given by 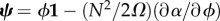 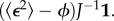 Note now that the vector **1** is an eigenvector of *J* with eigenvalue *α*. It follows that **1** is also an eigenvector of *J*^−1^ with eigenvalue 1/*α*, and we can, therefore, simplify *J^−^*^1^**1** to (1/α)**1**. The sEMRE then becomes a vector with every entry the same, so we write the scalar *ψ* as
3.12
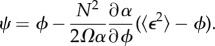
It remains therefore only to find the value of the quantity 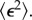 This is given by the first entry of the matrix *C* which is defined by the Lyapunov equation given in equation (2.11). We note that by the symmetries of the system, both *J* and *C* must be symmetric, circulant matrices [31], which implies that *JC* = *CJ*^T^, and therefore, the Lyapunov equation can be simplified to *C* = −(1/2)*J*^−1^*D*.

The block circulant structure of *J* allows us to find an analytical formula for 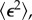 which is equation (A 25) in appendix A. Combining equation (3.12) with equation (A 25), we get a formula for *ψ*
3.13
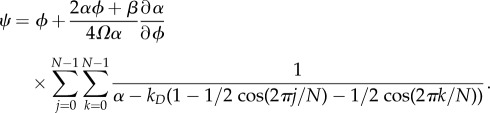
Equivalent formulae for one- and three-dimensional topologies are given at the end of appendix A. In appendix B, we show that the formula (3.13) can be greatly simplified when *N* is large compared with one
3.14
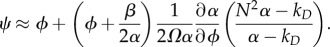


It can also be shown from Jensen's inequality that this approximation is a lower bound for *ψ* determined from equation (3.13) (see appendix B), although as we shall see it is typically numerically nearly indistinguishable from *ψ*. Equation (3.14) can be written in a particularly informative way to distinguish the contributions from the EMRE and the sEMRE
3.15
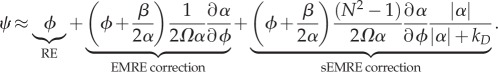
Note that the sign of the sEMRE correction is guaranteed to be the same as that of the EMRE correction, because the former is a positive multiple of the latter. Note also that the spatial correction term is proportional to an MM term, |*α*|/(|*α*| + *k_D_*), with the absolute values arising from the guaranteed negativity of *α*. This term monotonically increases from 0 to 1 as the diffusion rate *k_D_* decreases implying that the absolute difference between the stochastic and deterministic solutions 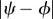 increases with decreasing diffusion coefficients. Note also that the difference is proportional to the Hessian of the REs 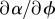 and hence it is non-zero only if there is at least one bimolecular reaction. The equations derived in this section generally apply to systems with mass-action kinetics; however, systems with any type of rate (including Hill-type and MM-type rates) are also compatible with the sEMRE. In appendix C, we show that the sEMRE for such systems is simply given by equation (3.13) but with an extra added term, and in §5, we study an example system with MM-type rates.

Hence summarizing, our result shows that the steady-state mean concentrations for a spatially homogeneous one-species system generally depend on the diffusion coefficients. In contrast, the spatial deterministic solution *ϕ* and the reaction–diffusion PDEs have no such dependence. This diffusion dependence is therefore a stochastic effect.

Of course, one could also obtain the sEMREs for an effective one species system without the condition of spatial symmetry, but then an explicit solution in closed-form will be difficult, if not impossible to obtain. The diffusion dependence of the mean concentrations in each voxel will then have two components, one stemming from the spatial heterogeneity of the rate constants or diffusion coefficients, and one stemming from intrinsic noise as found above. The steady-state solution of the spatial REs will only be able to capture the first component and hence the diffusion dependence of the concentrations according to sEMRE will be different from those of the deterministic approach, even in the absence of spatial symmetry. Using a completely analogous approach, one could also derive sEMREs for a multi-species system, but once again closed-form steady-state solutions will be difficult to obtain. A detailed discussion of a numerical solution of the time-dependent sEMRE for a multi-species system (allowing for space-dependent diffusion and reaction rates, as well as general topologies) can be found in appendix F.

In the rest of this article, we apply our results to two examples of simple gene regulatory networks under the condition of spatial symmetry. We confirm our results by comparison with RDME and BD simulations.

## 4. Application: gene regulatory circuit in a single cell

**Figure 2.**
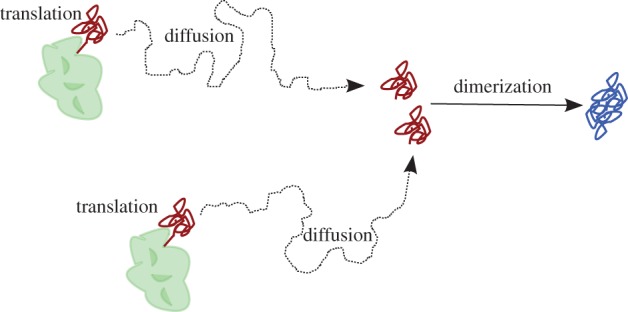
Schematic diagram of protein dimerization model (4.1). Uniformly distributed ribosomes (green) translate proteins (red). These diffuse in the cytosol until a pair combines to form a dimer (blue).

In this section, we apply the sEMRE to a simple model of protein production and dimerization in a single cell, shown schematically in figure 2.

Ribosomes (green) translate proteins (red) which diffuse through the cytosol until a pair meets and they dimerize into a product. We do not model the ribosomes explicitly, rather knowing that ribosomes are numerous (in the thousands per cell) and known to be uniformly distributed for some types of cells (for example for *Escherichia coli* in the exponential phase, ribosomes are spread uniformly around the nucleoids [32]). We therefore roughly model the translation of proteins by ribosomes via a zeroth-order reaction at all points inside a cell. Hence, the system, in figure 2, is approximated by the reaction scheme
4.1
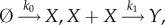
where *X* is the protein and *Y* is the dimer.

In the following, we describe a BD algorithm for continuum space simulations of the protein *X* in the above system and compare the results of these simulations with the sEMRE approximation of the RDME derived in the previous section. Note that we ignore *Y* in our simulations because it has no influence on the proteins which produce it. As we will show, BD simulations verify our theoretical result: generally, the steady-state mean protein concentration has a strong dependence on the diffusion coefficient.

### 4.1. Brownian dynamics

BD models the diffusion of solute particles in continuum space as shown in figure 1. The boundaries of the area are periodic, such that a particle which crosses a boundary appears at the opposite boundary. Reactions between two particles occur with some non-zero probability if the particles overlap. For single-cell modelling, particularly if the cells are prokaryotic and have no intracellular structures, there is not a natural length scale for which solute particles can be considered to be well mixed. In this case, BD is a more accurate description of real reaction–diffusion processes than the RDME.

In order to compute mean concentrations from BD, one long simulation is performed (much longer than the time to reach equilibrium), and the mean number of particles is simply the average number over that time. Particles are circles with radius *r* and have a diffusion coefficient, *D*. The area of space is *Ω*. The steps of the algorithm are then as follows:

(1) Choose a reaction probability per unit time, *p* and a time interval Δ*t*. Set time counter *t* = 0. Generate an Exponential(1/*k*_0_*Ω*) random number *τ*.
(2) Add a normal random number with zero mean and variance 2*D*Δ*t* to each particle coordinate. Add Δ*t* to *t*.
(3) For each pair of intersecting particles (when the distance between the particle centres is less than 2*r*), generate a uniform random number. If it is less than *p*Δ*t*, remove both particles.
(4) If *t* > *τ*, then add a new particle at a uniformly distributed point in space. Generate an Exponential(1/*k*_0_*Ω*) random number and add it to *τ*.
(5) Repeat steps 2–4 until the desired time has elapsed.

This algorithm is an example of the Doi model of BD [33]. A popular alternative is the Smoluchowski model [34] in which particles react immediately when their reaction radii overlap, which corresponds to the above algorithm with 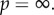 There are two reasons why we chose not to use the latter method. First, we expect the CME to agree with BD for large diffusion coefficients, but in the CME, the probability of a reaction in a time Δ*t* is proportional to Δ*t* [35]. We therefore use *p*Δ*t* to ensure that BD has the same property. The second reason for using the quantity *p*Δ*t* is that, in reality, dimerizations only occur if molecules approach each other with the correct relative orientations and the correct kinetic energy [36]. In BD, we do not consider either orientation or kinetic energy, and so instead we approximate the molecular physics by saying that a collision leads to a reaction with a probability strictly less than 1.

### 4.2. Parameter choices for comparison between models

To compare the sEMRE with BD, we will need to relate the various parameters used by each of them, which we do in this section.

The value of *p* that we choose for BD is given (in two dimensions) by the simple equation *p* = *k*_1_/2*πr*, and a derivation of this result is given in appendix D. This choice guarantees that in the limit of well-mixed conditions, the rate at which the dimerization occurs in the BD description agrees with that given by the bimolecular propensity in the CME. The rate of the birth process, *k*_0_, is the same in all models.

The choice of relation between *D* and *k_D_* is given by the equation 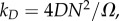 which is valid in two dimensions. This result can be derived either from Fick's law or from a mean first passage time approach [37].

The final choice of parameters for comparison is the number of voxels *N*^2^, given that we choose our system size *Ω* to be 1 and particle diameter to be 1/20. There is no obvious choice of voxel size, except that the voxel should be larger than a molecule, that is, *N* < 20. Several authors have proposed bounds for a correct choice of *N*; see [37] for a summary.

### 4.3. Comparison of Brownian dynamics with spatial effective mesoscopic rate equations

**Figure 3.**
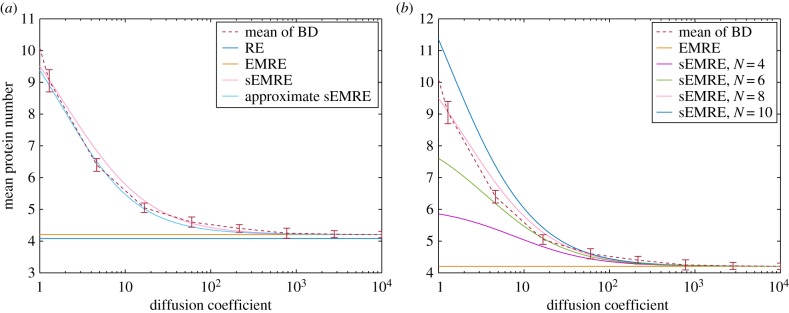
The mean steady-state molecule number of protein *X* in system (4.1) as a function of the diffusion coefficient *D*. (*a*) We compare the result of two-dimensional BD simulations in steady-state conditions (dashed red) with the sEMRE, RE and EMRE approximations of the RDME on a two-dimensional *N* × *N* grid with *n* = 8. The RE corresponds to the deterministic spatial approximation of the RDME, the EMRE corresponds to the deterministic approximation of the CME plus a correction to take into account a finite system size *Ω*, whereas the sEMRE corresponds to the EMRE plus a correction to take into account finite diffusion coefficients *D*. The RE is given by the first term in equation (4.4), the EMRE by equation (4.4) with 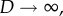 the sEMRE by equation (4.3) and the approximate sEMRE by equation (33). (*b*) Comparison of BD simulations (red) with sEMRE equation (4.3) with *N* = 4 (purple), *N* = 6 (green), *N* = 8 (pink) and *N* = 10 (blue). Parameter values are *k*_0_ = 1000, *k*_1_ = 30, *Ω* = 1, molecule diameter = 1/20 and Δ*t* = 10^−5^. Error bars are the standard deviation of 10 estimates of the mean protein number, each computed from a time average of a BD trajectory of length 10^4^ iterations.

Under the RDME, the reaction system (4.1) takes the form
4.2
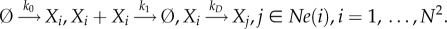
The sEMRE formula given by equation (3.13) can be applied specifically to the system (4.1). We find that it gives the formula
4.3
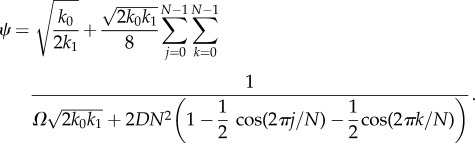
Alternatively, we can use the approximate formula given by equation (3.14)
4.4
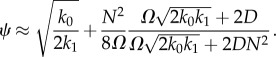
In figure 3*a*, we compare the steady-state mean concentrations obtained from BD simulations with the sEMRE formula for *N* = 8. The sEMRE agrees well over the whole range of diffusion coefficients, and the approximate formula is also an excellent approximation. The RE and EMRE cease to be good estimates at roughly *D* = 100. In figure 3*b*, we show that, for small enough diffusion coefficients, the choice of *N* is fundamental to the accuracy of the sEMRE. When *D* < 10, only the sEMRE with *N* = 8 gives an accurate estimate of the mean values of BD; however, the sEMRE for any *N* gives good estimates for *D* > 10. This is in agreement with the fact that the RDME agrees with BD only for intermediate voxel sizes (not too big and not too small); detailed discussions of this fact can be found in [23,37]. Note that the dependence of the accuracy of sEMRE with the choice of *N* stems from the RDME which sEMRE approximates. However, this is not of much concern, because for all *N*, sEMRE captures the correct qualitative behaviour (the monotonic increase of the steady-state mean concentrations with decreasing diffusion coefficient) that we observe from BD simulations.

### 4.4. Spatial effective mesoscopic rate equations of the volume-excluded reaction–diffusion master equation

The sEMRE is derived for the standard RDME, but can equally be applied to alternative RDMEs. One example is the recently introduced volume-excluded RDME (vRDME) [38]. The vRDME is a crude model of molecular crowding [39] which is known to agree well with BD, and which assumes that each particle occupies a fixed, non-zero volume and thereby places an upper bound on the number of particles in the system. This is done by shrinking the voxel size to be approximately equal to the size of a single particle. Voxels can then either be empty, or else contain exactly one particle. Bimolecular reactions take place between neighbouring voxels, and a particle can diffuse only if a neighbouring voxel is empty. This is achieved by a introducing an ‘empty space particle’, a dummy species which occupies a voxel if it is empty.

For the dimerization example, the vRDME replaces the reaction system given by (4.2), with the following:
4.5
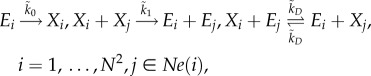
where *E_i_* represents an empty space particle in voxel *i*. Note that reaction rates are given as 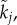 because they will, in general, take a different numerical value from the *k_j_* used in the RDME.

The sEMRE for this system is derived in appendix E:
4.6
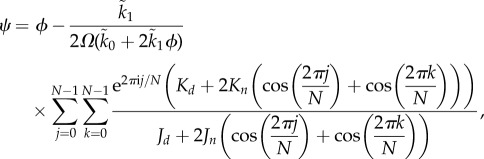
where 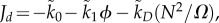 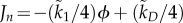 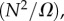 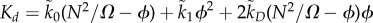 and *K_n_* = 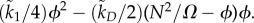


**Figure 4.**
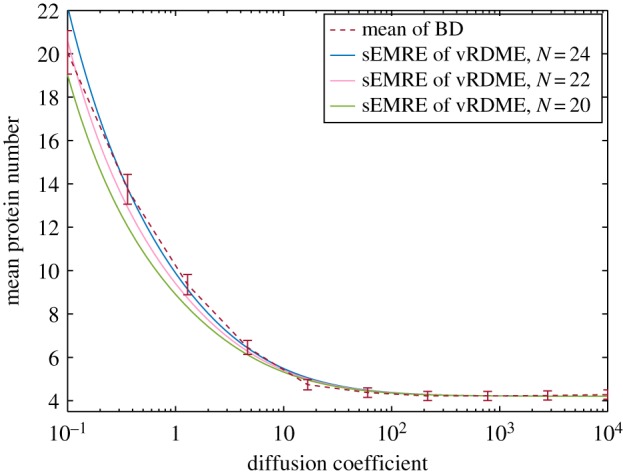
The sEMRE of the vRDME system defined in equation (4.6) as a function of diffusion coefficient *D*. The natural choice of *N* is the total area of space divided by the area of one (circular) particle, in this case *N* = 22 (pink line) which passes through every error bar of the BD simulations (red line). Small changes in the choice of *N* (blue and green lines) do not significantly affect the predictions of the sEMRE. Parameter values are *k*_0_ = 1000, *k*_1_ = 30, *Ω* = 1, molecule diameter = 1/20 and Δ*t* = 10^−5^. The parameters 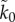 and 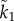 are chosen so that the vRDME and RDME agree in the limit of fast diffusion. See appendix E for details.

A significant advantage of the vRDME over the conventional RDME is that the choice of *N* is automatic in the former case: we simply choose an integer *N* such that 1/*N*^2^ is approximately the volume fraction occupied by a single (circular) particle. The benefits of this can be seen in figure 4, where we plot the sEMRE in equation (4.6) against BD simulations. The particle diameter used in BD is 1/20, which suggests choosing 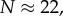 and indeed, the sEMRE for this *N* passes through every error bar down to *D* = 10^−1^, which is an order of magnitude lower than that plotted in figure 3. We also show the sEMRE with *N* = 20 and *N* = 24, which both give good approximations to the BD simulations, demonstrating that *N* only needs to be approximately correct to give accurate results.

Note that the BD simulations for this example are slightly different, because we are trying to model volume exclusion. The only difference between this BD and the algorithm described in §4.1 is in point 3 of the algorithm. In this case, we would add ‘if the uniform random number is greater than *p*Δ*t*, subtract Δ*t* from *t* and return to 2’.

## 5. Application to a system of intercommunicating cells

Everything derived thus far generally applies to systems with mass-action kinetics; however, systems with any type of rate (including Hill-type and MM-type rates) can also be analysed using the sEMRE approach. As we show in appendix C, the sEMRE for such systems is simply given by equation (C 6), which is nothing more than equation (3.13) with an extra added term. In this section, we therefore apply our results to a more complex system that can be reduced to an effective single-species system with non-elementary rates.

**Figure 5.**
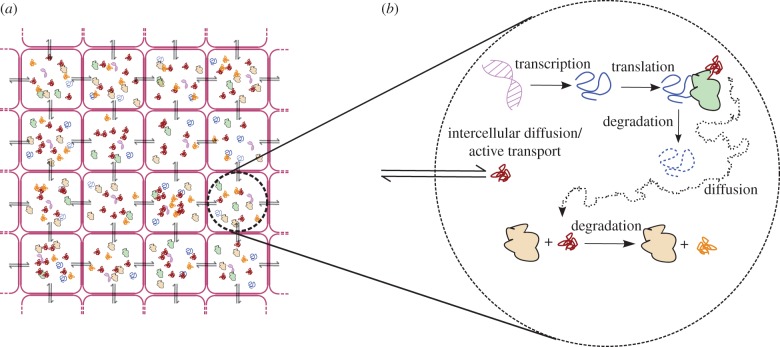
Schematic diagram of a tissue of close-packed cells, each with a gene regulatory network inside and communicating via chemical exchange. (*a*) Cells are organized as a two-dimensional grid. Proteins are created inside a cell and can move between neighbouring cells by active transport or diffusion. (*b*) Detailed picture of the reactions involved in each cell—see reaction scheme (5.1). DNA (pink) transcribes mRNA (blue) which translates proteins (red) via a ribosome (green, not modelled explicitly) until the mRNA degrades. These proteins diffuse until they bind to an enzyme (peach) which modifies them into a product (orange) through a standard MM reaction. Proteins can also move between neighbouring cells by diffusive or active transport. We assume, in our calculations, that intracellular diffusion is fast, so that well-mixed conditions occur in each cell.

In particular, we consider the system illustrated in figure 5, a tissue of identical cells arranged in a grid-like formation. Inside each cell, an mRNA molecule, *M*, is transcribed with rate *h*_0_ and degrades with rate *h*_1_. It translates a protein, *X*, with rate *h*_2_. This protein is consumed by an enzyme, *E*, which forms a complex, *C*. This can either unbind back to the protein with rate *h*_4_ or else convert the protein to a product, *P*, with rate *h*_5_. Proteins can also move between neighbouring cells by a combination of active transport and diffusion. A clear difference between this example and the one described in figure 2 is that here each voxel represents a single well-mixed cell, rather than a small region of a cell. Furthermore, the choice of *N*^2^ now has a clear physical significance: it is simply the number of cells in the tissue. The system in each cell can be defined in terms of the reactions
5.1
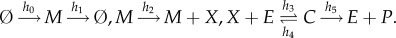
The well-mixed, non-spatial version of this system has been studied in detail in [30,40], whereas here we study the spatial version using the sEMRE approximation.

It is known that in bacteria and budding yeast, the mRNA lifetime is generally considerably shorter than that of the protein. Under such conditions, it has been shown that protein synthesis occurs in geometrically distributed bursts [10]. We therefore consider the overall birth process of a protein (transcription plus translation) to be effectively modelled by the single reaction 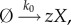 where *z* is a geometrically distributed random number with mean 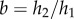 and 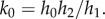 Furthermore, the enzyme-driven catalysis of *X* can be written as a simple first-order decay 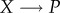 with an effective MM-type propensity 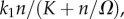 where *n* is the number of molecules of *X*, 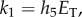 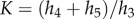 and *E*_T_ is the total enzyme concentration. This approximation is accurate, in a stochastic setting, in the rapid equilibrium limit 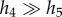 [41,42]. Hence, it follows that reaction scheme in each cell (5.1) can be adequately described by the single-species system

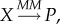

5.2
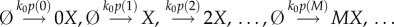
where the first line describes nonlinear degradation via an MM propensity and the second line describes bursty protein production. Note that 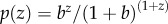 is the probability distribution of a geometric random variable *z* (the burst size) with mean *b*. This effective representation for the input reaction has been previously used to study the effects of bursts on the oscillatory properties of downstream pathways [43].

Now, we compute the sEMRE for this reduced system. We label the enzyme catalytic reaction as reaction 1, and the reaction producing *z* bursts as reaction 2 + *z*. The stoichiometric matrix *S* and rate vector ***f***(*ϕ*) are then defined by
5.3
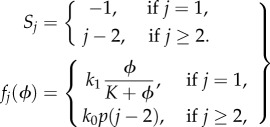
where *ϕ* is the deterministic concentration of *X*. From these, one can compute the REs, the Jacobian *α* and the diffusion matrix *β* using the definitions given previously. The steady-state RE solution is given by 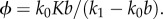 The Jacobian is 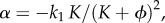 and the diffusion matrix is 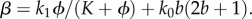 These formulae can be plugged in equation (C 6) to obtain the sEMRE
5.4
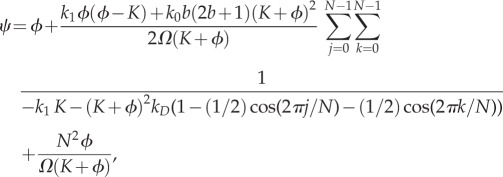
or using the approximate formula given by equation (C 7),
5.5
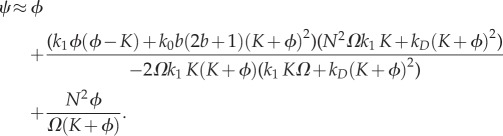
Note that *ψ* is here to be interpreted as the approximate concentration of protein in each well-mixed cell, taking into account the noise from reactions inside each cell and from protein exchange between cells. We remind the reader that the tissue has *N*^2^ identical cells and total area *Ω*, protein is generated in each cell by a gene regulatory network with parameters *b*,*k*_0_,*k*_1_,*K* and is exchanged at a rate *k_D_* with neighbouring cells via diffusion or active transport.

**Figure 6.**
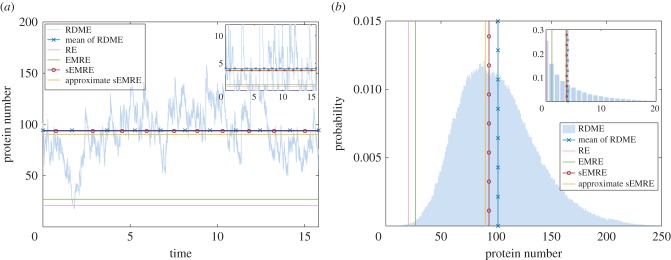
(*a*) Typical global trajectory (across all tissue) of the RDME (pale blue) describing the non-reduced system (5.1) in each cell (voxel) and protein exchange with rate *k_D_* between cells. The mean value of the RDME calculated over the trajectory is shown as blue crosses. Further lines give the RE (pink), EMRE (green), sEMRE (red circles) and approximate sEMRE (yellow). Inset: local trajectory in a particular cell (voxel). (*b*) Steady-state distribution of the RDME (pale blue histogram), with solid lines showing the RDME mean (blue crosses), RE (pink), EMRE (green), sEMRE (red circles) and approximate sEMRE (yellow). Inset: local distribution in a particular cell. Parameter values are *k_D_* = 100, *h*_0_ = 2, *h*_1_ = 1, *h*_2_ = 4, *h*_3_ = 30, *h*_4_ = 30, *h*_5_ = 5, *E*_T_ = 10, *Ω* = 10, *N*^2^ = 25.

To verify our formulae, we carried out RDME simulations. In figure 6*a*, we plot a typical steady-state trajectory of total protein numbers (sum over all voxels) obtained from the RDME describing the reduced system (5.2) in each cell (voxel) and protein exchange with rate *k_D_* between cells. This is compared with various estimates of the mean concentrations. The RE (pink) and EMRE (green) both give remarkably bad estimates of the mean concentration of the RDME (blue crosses). On the other hand, the sEMRE (red circles) and approximate sEMRE (yellow) both give a good approximation, only a few molecule numbers away from the true mean. In the inset, we show the local protein trajectory, i.e. that in a single cell (voxel) of the tissue. Again, the RE and EMRE give poor estimates, whereas the sEMRE and approximate sEMRE are in good agreement with the mean of the RDME.

In figure 6*b*, we plot the typical steady-state probability distribution for protein numbers from the RDME, computed with a time average over 10^6^ iterations (blue histogram). This is compared with various estimates as in figure 6*a*, once again showing the accuracy of sEMRE. In the inset, we show the local distribution of protein numbers in a single cell, again the RE and EMRE give inaccurate estimates, whereas the sEMRE and approximate sEMRE agree well with the true mean. Hence, RDME simulations verify the accuracy of the sEMRE approximation, and in particular, the strong dependence of the steady-state mean concentrations on the diffusion coefficients which is not captured by the deterministic spatial RE models. Note that the slight difference in RDME means between figure 6*a,b* is due to different RDME trajectory lengths used in generating the two plots.

## 6. Discussion

In this paper, we have shown that the mean concentrations of a single-species reaction–diffusion system in equilibrium generally depend on the diffusion coefficients: this contradicts the popular reaction–diffusion PDEs, and is therefore a stochastic effect. We obtained an approximate formula for the steady-state mean concentrations of an effective one species system according to the RDME, the conventional stochastic spatial description of kinetics. This expression is a sum of three components: a term given by the deterministic REs, and two terms that correct the solution of the latter to take into account a finite compartment size (or equivalently finite molecule numbers) and finite diffusion coefficients. We verified this result by applying it to two simple models of gene regulatory systems and comparing our approximate formula with RDME and BD simulations. In particular, the comparison with BD shows that the predicted noise-induced dependence on the diffusion coefficients in steady state is not because of the artificial spatial lattice of the RDME but rather a genuine phenomenon.

An intuitive explanation of the effect is as follows. Let 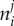 be the molecule number of species *i* in voxel *j*. The average rate at which a bimolecular reaction occurs in a voxel *j* of space is necessarily proportional to the average of the product of the local molecule numbers of the two species involved in the reaction 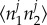. Hence, we can write the local average rate as the sum of two terms: 
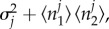
 where 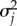 is the covariance of fluctuations in voxel *j*. Clearly, the second term is the deterministic contribution to the average rate as given by the spatial REs. The first term is the stochastic contribution to the average rate. Now, two different processes lead to a non-zero covariance of fluctuations in a voxel: (i) the variability in the time between reaction events occurring inside the voxel, i.e. intrinsic noise, and (ii) particle exchange between neighbouring voxels of space stemming from local diffusion. Because the steady-state mean concentrations depend on the average rates of reaction, it follows that they must depend on both the size of intrinsic noise owing to finite copy numbers (this is the EMRE correction in equation (3.15)) and on the size of diffusion coefficients (this is the sEMRE correction in equation (3.15)).

We finish by noting that, although in this paper we focused on time-independent and spatially symmetric solutions of sEMRE, these two assumptions are only needed to obtain compact closed-form formulae and they are not a limitation of the formalism. Without these assumptions, the set of coupled ordinary differential equations constituting sEMRE can be solved numerically for any number of species and will be advantageous from a computational point of view because unlike RDME (or BD) simulations, the solution of sEMRE does not require ensemble averaging (see appendix F). In particular, the relaxing of spatial symmetry will allow the modelling of stochastic reaction kinetics in tissues composed of cells exhibiting a high degree of cell-to-cell variation. Just as we obtained equations for the mean concentrations, one can also obtain ordinary differential equations for the higher moments in each voxel of the RDME. Hence, we anticipate that extensions of the present formalism along the aforementioned lines may greatly enhance our understanding of spatial and stochastic reaction kinetics in various biological contexts.

## Appendix A. Detailed derivation of spatial effective mesoscopic rate equations

For two-dimensional topologies, *J* and *D* are the block matrices given by equations (3.9) and (3.10), which have the structure

A 1

A 2

where

A 3

A 4

and  is the identity matrix. We seek the first element of the matrix *C* defined by

A 5

Because *J* is block circulant, its inverse is also, and we can write it as

A 6

Then, *C* will be given by

A 7

The defining equations for the relevant *B_i_* are [44]

A 8

Because  is a circulant matrix, we can write its inverse as

A 9

where   is diagonal such that

A 10

Therefore,

A 11

where

A 12

Similarly,  and  where

A 13

and

A 14

*F_N_* has the structure

A 15

So the contributions to  which is the first entry of the matrix *C*, will be obtained by substituting equation (A 8) into equation (A 7). The first contribution will be proportional to

A 16

A 17

A 18

A 19

The second contribution will be proportional to

A 20

A 21

A 22

Similarly, the third contribution will be proportional to

A 23

We can combine the second and third contribution into

A 24

The final result is that

A 25

Therefore, combining equation (A 25) with equation (3.12), we can explicitly write a formula for *ψ*

A 26

Similarly, for one dimension, it can be shown that

A 27

whereas for three dimensions

A 28

## Appendix B. Derivation of approximate spatial effective mesoscopic rate equations

The sEMRE in two dimensions is given by

B 1

This sum can be separated into two parts

B 2

The first part corresponds to the term *j* = 0, *k* = 0. The second part covers all the terms other than *j* = *k* = 0 (this is denoted by 0*). Now, we consider what happens in the limit of large *N*. The double sum can be approximated by an integral

B 3

This is equal to the expected value of the integrand under the uniform distribution

B 4

for i.i.d. uniform random variables *X*,*Y*. By Jensen's inequality, we have

B 5

We therefore have a lower bound for the sEMRE

B 6

This is precisely the approximation formula given in equation (3.14).

## Appendix C. General formulation of effective mesoscopic rate equation/spatial effective mesoscopic rate equation for elementary and non-elementary rates

In this appendix, we follow the generalized derivation of the EMRE in [45] and apply it to the sEMRE. For a system of reactions given by equation (2.1), the CME is defined as

C 1

where  is the vector of molecule numbers of species *X*_1_, … ,*X_N_, P*(***n***, *t*) is the probability that the system has exactly *n*_1_, … ,*n_N_* copies of species *X*_1_, … ,*X_N_*, respectively, at time *t*,  is the step operator which replaces *n_i_* with *n_i_* + *x*, and  is the microscopic propensity function of reaction *j*, i.e. the probability per unit time that reaction *j* will happen if the system is in state ***n***. Now, define *a_j_* as

C 2

which is simply the microscopic propensity  evaluated at the deterministic concentration. Expanding this as a power series in *Ω*^−1^, we can write

C 3

where  are the coefficient functions. The zeroth coefficient  is equal to the macroscopic reaction rate *f_j_* defined in equation (2.5). The first coefficient  can be used to define the quantity :

C 4

The REs are given by equation (2.5). The time-dependent EMRE is then given by equation (2.9) and the Lyapunov equation (2.8) together with the mean-covariance coupling vector

C 5

Note that for elementary reactions, one can show that  which precisely recovers equation (2.7).

This generic EMRE formulation can be applied to the case of one species involved in a set of elementary or non-elementary reactions together with diffusion reactions on a lattice and this constitutes the generic single-species sEMRE. Hence, the latter is essentially obtained by the same arguments as in §3.2, however, using equation (C 5) rather than using equation (2.7). This gives the result

C 6

Note that given the spatial homogeneity of the system, all the  will take the same value, *D*^(1)^. Equation (C6) generalizes equation (3.13).

We can also obtain an approximate formula which generalizes equation (3.14):

C 7

Note that MM and Hill-type reactions do not contribute to *D*^(1)^, and therefore, the sEMRE applied to system (5.2) is equation (C 6) with *D*^(1)^ = 0.

## Appendix D. Relation between the bimolecular reaction rate and the probability of reaction given collision

For a system in a two-dimensional area *Ω*, we expect the CME to agree with BD when diffusion is ‘fast’, which really means in the limit  Generally, at each BD time step, particle positions are updated by a normal random number with mean zero and variance 2*D*Δ*t*; but in the limit of fast diffusion, this normal distribution will have infinite width. Because our topology has periodic boundary conditions, particles will be uniformly distributed at each time step. Particles collide if they intersect: for two particles with radii *r*_1_ and *r*_2_, a collision occurs if the particle centres are within *R* = *r*_1_ + *r*_2_ of each other. The probability of a collision between two particles in a single time step is therefore the probability that a uniformly distributed point falls within a circle of radius *R*. That is

D 1

Now, suppose that we have a system involving a bimolecular reaction  for some species *X*_1_ and *X*_2_ with radii *r*_1_ and *r*_2_, respectively, and molecule number *n*_1_ and *n*_2_, respectively. There are, therefore, *n*_1_*n*_2_ possible pairings of reacting particles. In a given time step Δ*t*, the probability that a given pair collides is  and in BD, the probability that a reaction results in a collision is *p*Δ*t*. The probability that a given pair reacts is therefore

D 2

Assuming independent collisions, the total *number* of reactions that occur in a given time step is then given by a Binomial  distribution. By definition, the probability of *m* reactions occurring a Δ*t* is then given by

D 3

The probability of 0 reactions is then

D 4

and the probability of 1 reaction is

D 5

All further terms are *O*((Δ*t*)^2^). If Δ*t* is chosen small enough, then we can ignore terms of *O*((Δ*t*)^2^). Although many collisions may occur in a single time step, Δ*t* is chosen small enough, so that at most one of these can result in a collision. At the CME level, the reaction  occurs with a rate which implies that the probability that the reaction occurs in a time step Δ*t* is  Equating this expression with equation (D 5), it follows that

D 6

In the special case where the bimolecular reaction happens between particles of the same type, i.e.  we instead have  possible distinct particle pairings, and a CME rate of  The relation therefore becomes

D 7

## Appendix E. Derivation of spatial effective mesoscopic rate equation for volume-excluded reaction–diffusion master equation

We can write down the RE for *X*^(*i*)^ in the system (4.5):

E 1

where *ϕ_i_* is the concentration of *X* in voxel *i*. The first term corresponds to the birth reaction, the second term to the four possible dimerizations (each with a different neighbouring voxel), and the third and fourth to diffusions into and out of voxel *i*, respectively. Note that  corresponds to the concentration of empty space in voxel *i*, *E_i_*. This is because each voxel can contain either *E* or *X*, and hence, there exists a conservation law in each voxel. Also note that the factor of  is due to the fact that the dimerization can occur between *X_i_* and four distinct neighbours; by dividing by 4, we ensure that the total dimerization rate for a given *X_i_* is  By the spatial symmetry of the system, all the *ϕ_i_* are equal, say, *ϕ*. Because the diffusion terms in equation (E 1) cancel under this assumption, *ϕ* is simply the solution of a quadratic

E 2

We can then write down the entries for the Jacobian matrix *J* and diffusion matrix *D*, analogously to the method used in §3.2. For the Jacobian, we have

E 3

For the diffusion matrix, we have

E 4

The method of computing the sEMRE of the vRDME is now identical to the sEMRE of the RDME, albeit with different values of *J* and *D*. Following the same method, we obtain the analytical expression for the sEMRE

E 5

where *D_d_* and *D_n_* are the diagonal and neighbouring elements of *D,* respectively, and similarly for *J_d_* and *J_n_*. In other words,      Equation (E 5) is precisely equation (4.6) in the main text.

The relationship between the parameters  of the vRDME and the parameters *k_j_* of BD and the RDME requires careful attention. A detailed discussion can be found in [38]. Here, we choose parameters such that the vRDME and BD generally agree when diffusion is fast. To do this, we compare the RE of the vRDME with the RE of the CME (or equivalently the RDME) which we know is the fast-diffusion limit of BD. The RE are convenient, because neither of them depend on the diffusion rate  or *k_D_*. It follows that we do not need to worry about rescaling this parameter; therefore, we set  The steady-state RE solution of the CME is given by  whereas the RE solution of the vRDME is *ϕ^V^* given by equation (E 2). We therefore need to choose values of  and  which allow the concentrations to agree. In this paper, we have chosen

E 6

## Appendix F. Spatial effective mesoscopic rate equations for systems with more than one species

In this paper, we have focused on systems which either have one species, or else which can be satisfactorily reduced to a system with one effective species. This is because it is only for such systems that the Jacobian and diffusion matrices *J* and *D* are block circulant matrices, and this fact is essential for deriving analytical expressions for the sEMRE. However, the sEMRE can be calculated numerically for any system with any number of species. Furthermore, the sEMRE is not only applicable to systems of a two-dimensional periodic grid. We therefore here consider a system of *N* (not *N*^2^) voxels which can be connected in any way. Consider a general system of reactions with *M* species, *N* voxels and *R* reactions, where the *j*th reaction has the form

F 1

where  represents species *X_k_* in voxel *i*, and  is the rate of reaction *j* in voxel *i*. This system is coupled with a set of diffusion events

F 2

where  is the rate at which *X_k_* diffuses out of voxel *i*, and *Ne*(*i*) is the set of voxels neighbouring *i*. This description is completely general, because reaction and diffusion rates can vary between voxels: spatial heterogeneity is therefore permitted.

The deterministic concentration of  is denoted  and the vector of concentrations is given by

F 3

The stoichiometric matrix is now  where  is the sum of the number of neighbours of each voxel, i.e. the total number of distinct diffusion events which can occur (if each voxel has four neighbours this would be 4*N*). The first *RN* columns of *S* correspond to reaction events and so  for the relevant reaction in this case. The last *cM* columns of *S* correspond to diffusions, and so have entries 1 and −1 for the species created and destroyed respectively, and zeros otherwise. The macroscopic rate vector  now has its first *RN* entries corresponding to reaction rates, and the last *cM* entries corresponding to diffusion events. The spatial RE solution in a time-dependent setting is given by

F 4

or else in steady state by

F 5

The Jacobian  is defined as

F 6

and the diffusion matrix  is defined as

F 7

where both equations hold for both time-dependent and steady-state descriptions. The variance–covariance matrix  is defined by

F 8

in the time-dependent case, or else

F 9

in the steady-state case. The covariances are then given by  so that the vector  is defined as

F 10

which holds in both the time-dependent and steady-state cases. Note that this form for ***Δ*** assumes mass-action kinetics. For a discussion of non-mass-action kinetics, see appendix C. The sEMRE is finally given by

F 11

in the time-dependent case, or else by

F 12

in steady state. Note that this recipe can equally be used for systems without spatial homogeneity (such as a birth process which takes place in only one voxel). The most significant computational cost is incurred by the solution of the Lyapunov equation (F 9). This process is at worst *O*(*N*^3^*M*^3^), but dramatic speed increases are likely, because *J* is a sparse matrix [46]. In contrast, the SSA scales with the total number of reaction and diffusion events per unit time, and furthermore, typically requires many ensemble averages to ensure meaningful results. The sEMRE avoids ensemble averaging and hence a direct comparison of computation time cannot really be made. However, because the total number of reaction and diffusion events per unit time increases with the number of molecules in the system [47], it is likely that the sEMRE is much faster than the SSA, provided that the number of molecules is not too small.
